# Quantum Revivals in Curved Graphene Nanoflakes

**DOI:** 10.3390/nano12121953

**Published:** 2022-06-07

**Authors:** Sergio de-la-Huerta-Sainz, Angel Ballesteros, Nicolás A. Cordero

**Affiliations:** 1 Physics Department, Universidad de Burgos, E-09001 Burgos, Spain; shuerta@ubu.es (S.d.-l.-H.-S.); angelb@ubu.es (A.B.); 2 International Research Center in Critical Raw Materials for Advanced Industrial Technologies (ICCRAM), Universidad de Burgos, E-09001 Burgos, Spain; 3 Institute Carlos I for Theoretical and Computational Physics (IC1), E-18016 Granada, Spain

**Keywords:** graphene, curvature, quantum revivals, DFT, phase transition

## Abstract

Graphene nanostructures have attracted a lot of attention in recent years due to their unconventional properties. We have employed Density Functional Theory to study the mechanical and electronic properties of curved graphene nanoflakes. We explore hexagonal flakes relaxed with different boundary conditions: (i) all atoms on a perfect spherical sector, (ii) only border atoms forced to be on the spherical sector, and (iii) only vertex atoms forced to be on the spherical sector. For each case, we have analysed the behaviour of curvature energy and of quantum regeneration times (classical and revival) as the spherical sector radius changes. Revival time presents in one case a divergence usually associated with a phase transition, probably caused by the pseudomagnetic field created by the curvature. This could be the first case of a phase transition in graphene nanostructures without the presence of external electric or magnetic fields.

## 1. Introduction

The experimental isolation of a single graphitic layer (now known as graphene) by means of the so-called “Scotch tape method” by Geim and Novoselov [1] has undoubtedly opened a new field in science. The proof is that in recent years, over 1% of all the scientific publications included in the Web of Science™ global citation database [2] are related to this one-atom-thick system.

The outstanding properties of this 2D material have led to many application proposals. To cite just a few of them: nanocomposites for bone tissue engineering [3], composites for multifunctional applications [4], lubrication [5,6], solar cells [7], ultracapacitors [8,9], batteries [10], sensors [11], catalysis [12], nanomedicine [13], fuel cells [14,15] or even energy harvesting [16].

Quantum revivals consist in the temporal periodic reconstruction of a wave packet in systems with a commensurable discrete spectrum [17]. They have recently attracted attention due to their possible interest in quantum devices [18,19,20,21]. Quantum revivals have been studied in infinite graphene under magnetic fields [22,23,24] or circularly polarized radiation [25], as well as in graphene rings [26] and graphene quantum dots [27].

One of the common misconceptions about graphene is that it is flat. In fact, Peierls instability [28,29] creates ripples in free-standing graphene [30,31,32,33,34,35]. Atomistic Monte Carlo simulations based on a very accurate many-body interatomic potential for carbon [36] gave ripples with a size distribution that peaked around 80 Å, in agreement with experiments that yield results in the 50–100 Å range [31]. Besides, when graphene is grown or deposited on a substrate, nanobubbles with diameters between 80 Å and 1000 Å appear [37,38,39,40,41].

Rectangular graphene flakes either free [42] or subjected to planar bending [43] have been analysed in the literature. Conical graphene rings in a magnetic field have also been studied, but only in the continuum limit approximation in the vicinity of the Dirac points and very far from the vertex [44,45]. Both in-plane and out-of-plane bending result in the appearance of a pseudo-magnetic field.

We present in this article the first (to the best of our knowledge) calculation of quantum revivals using Density Functional Theory (DFT) and use it to study the interplay between curvature and regeneration times in a non-planar graphene flake.

## 2. Materials and Methods

Since Wallace did the first calculation of the electronic properties of graphene using the tight-binding method [46], many theoretical models have been used to study this system: molecular mechanics, molecular dynamics, semiempirical methods, Density Functional Theory, Hartree–Fock (HF), post-HF including correlation, Monte Carlo simulations, hybrid methods, continuum models, etc. We have used the Density Functional Theory (DFT) formalism [47] within the Local Density Approximation (LDA) [48,49] as implemented in the Gaussian 09 [50] and Gaussian 16 [51] suites of programs. We have selected DFT for its balance between accuracy and computational effort, and have chosen LDA because it gives better results than gradient corrected approximations (GGAs) for graphitic systems [52,53,54] and because it has been previously used to successfully study the interaction between carbon nanostructures and several small molecules and atoms [55,56,57,58,59,60] as well as—very recently—graphene nanoribbons [61]. We have selected the 6-31G** basis set [62,63,64,65,66,67] that adds to the 6-31G set d-type and p-type Cartesian–Gaussian polarization functions and is commonly used for carbon nanostructures calculations.

**Figure 1:**
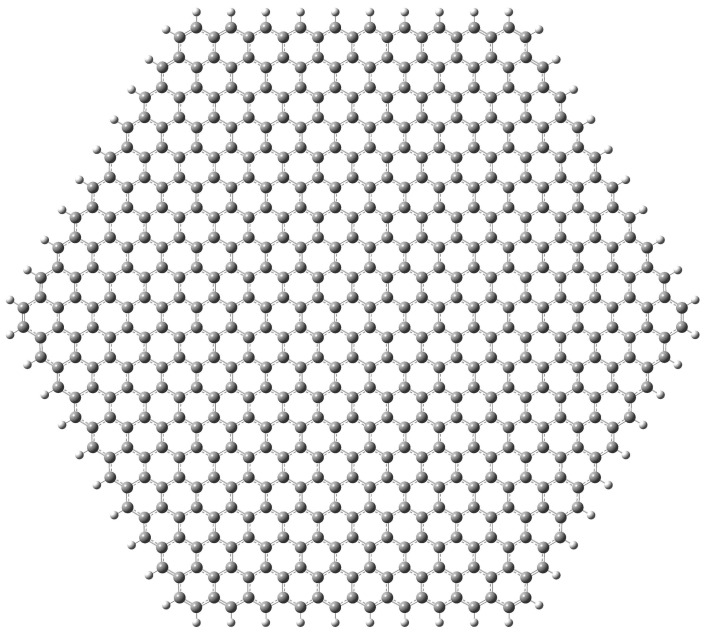
Graphene flake used in the calculations (image generated using GausView 6 [69]).

In order to avoid non-trivial magnetic ground states induced by asymmetric edge extensions [68] we have used the hexagonal flake with zig-zag borders passivated with hydrogen atoms shown in Figure 1. The size of the flake (10 concentric hexagonal rings comprising 600 C atoms and 60 H atoms with a separation of approximately 47 Å between opposite vertices) has been selected as a reasonable compromise between the size of experimentally measured ripples and computational cost.

**Figure 2:**
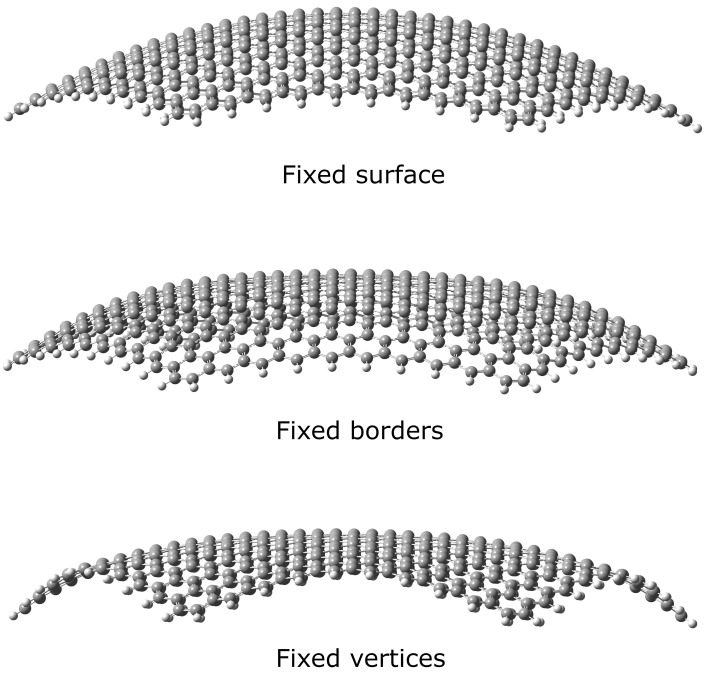
Optimized geometries for a (quasi-)spherical graphene flake of radius 40 Å (image generated using GausView 6 [69]).

We have studied (quasi-)spherical nanoflakes of different curvature radii with the three different bending possibilities depicted in Figure 2 for a radius of 40 Å: forcing all 600 carbon atoms to lie on a spherical surface (upper panel), fixing only the 60 border atoms on the surface and allowing the rest to relax (middle panel) and forcing only the 12 vertex atoms to belong to the sphere and not imposing any condition on the rest (lower panel). Since carbon atoms tend to be in a sp${}^{2}$ hybridization state, the nanoflake tends to flatten, as the restrictions are less demanding.

## 3. Results and Discussion

We have studied both curvature energy and quantum revivals in this nanostructure. The first one for checking if the macroscopic continuum limit model that predicts that curvature energy is proportional to the Gaussian curvature (i.e., the inverse of the radius squared) [70] holds at this scale. The second one for trying to find trends in regeneration times.

### 3.1. Curvature Energy

**Figure 3:**
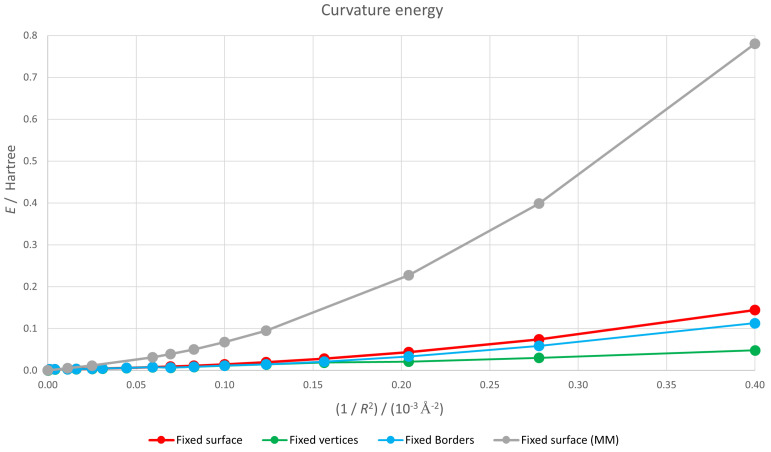
Energy of a (quasi-)spherical graphene flake as a function of its curvature. Flat configuration is taken as energy origin. Lines are merely guides for the eye.

We present in Figure 3 the dependence of the energy of the nanoflake *E* with the inverse squared radius of the sphere $\frac{1}{R^{2}}$. Since we have taken the flat configuration as the energy origin, this plot represents the curvature energy. For the case of a perfect spherical surface that corresponds to our fixed surface calculations (shown in red in the figure), $\frac{1}{R^{2}}$ is precisely the Gaussian curvature. For the fixed borders (depicted in blue) and fixed vertices (painted in green) cases, curvature is no longer constant, but we can take the value of $\frac{1}{R^{2}}$ for the fixed atoms as an estimate of the curvature for comparison purposes. Logically, for a fixed value of *R* the geometry with only the vertices fixed is energetically more favourable than that with the borders fixed and this in turn is more stable than the one with all the atoms forced to lie on a spherical surface. As expected, for the three geometries the curvature energy increases with curvature but the trend for the fixed surface case is somewhat unexpected. The macroscopic continuum result $E\propto \frac{1}{R^{2}}$ would lead to a straight red line, but that does not seem to be the case.

**Figure 4:**
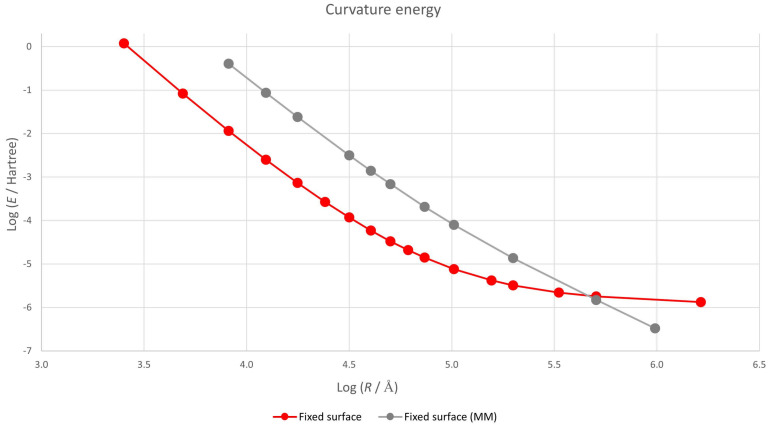
Log–log plot of the energy of a spherical graphene flake as a function of its curvature. Flat configuration is taken as energy origin. Lines are merely guides for the eye.

To check if the macroscopic result holds at least for low curvatures, we present in Figure 4 a log–log plot of the curvature energy versus radius.

(1) $$E=k\frac{1}{R^{2}}\;\Rightarrow \;\log E=\log k-2\log R,$$

and the red graph should present a constant slope equal to −2. This is clearly not the case and the continuum approximation is not valid for so small a flake.

This deviation from the continuum behaviour is undoubtedly due to the difference between the classical physics laws governing the macroscopic world and the quantum ones ruling at the nanoscale. This difference translates into two distinct aspects. The equilibrium geometry dictated by both sets of laws is different and, even for the same configuration, they lead to different energies. To check which one is more important in this case, we have made a series of non-self-consistent calculations with a hybrid Molecular Mechanics (MM)/DFT model. We have first used LDA calculations to determine a “semiclassical” Hooke potential for the C–C bond. To this end, we have used a graphene flake similar to the one in Figure 1, but with only 7 concentric hexagonal rings. We have changed the length of the central C–C bond and calculated the energy of the system for both stretched and compressed bond lengths using LDA. We have fitted the energies to a parabolic curve and determined a C–C pair potential for carbon nanoflakes. We have written an in-house code to determine the semiclassical equilibrium geometry for the spherical nanoflakes. This geometry is then used in a single-point calculation to determine the LDA energy of the nanoflake. We will label the results obtained by this procedure as “fixed surface (MM)”.

We have included in Figure 3 the energies resulting from this hybrid approach in gray. The energies are bigger that those corresponding to the self-consistent LDA calculation (in red) since, according to the variational principle, using a wave function different to that of the fundamental state leads to a higher energy. The greater the curvature, the bigger the energy increase.

In order to check if the continuum model is valid for this approach, we have also included the corresponding results in Figure 4. The graph is now nearly a straight line, but there is still some non-linearity. Equation (1) seems to be approximately valid, but the factor in front of logR depends slightly on *R*, adopting the form

(2) $$E=k\frac{1}{R^{n}}\;\Rightarrow \;\log E=\log k-n\log R.$$

**Figure 5:**
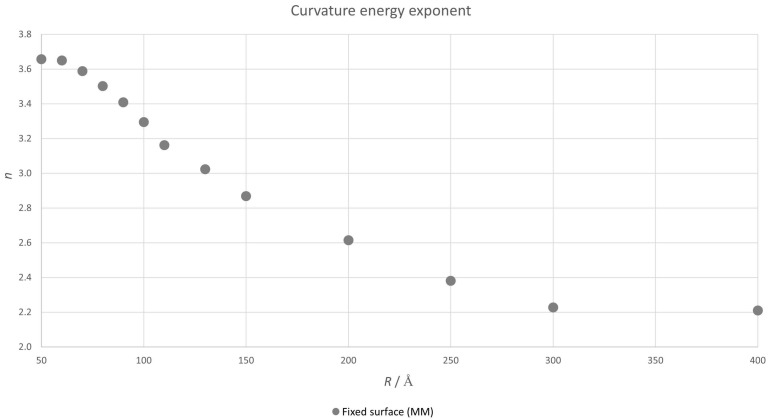
Value of *n* in Equation (2) as a function of the radius of the spherical carbon nanoflake calculated with the hybrid MM/DFT approximation.

We present in Figure 5 the value of *n* as *R* changes calculated using a 3-point finite differences method.

It is possible to fit the results in this figure using the simplest Padé approximant

(3) $$n=\frac{a_{0}+a_{1}R}{-1+b_{1}R}.$$

The result of this fitting is presented in Table 1.

We can use this Padé approximant to calculate the asymptotic behaviour of *n*.

(4) $$\lim_{R\to \infty }n=\frac{a_{1}}{b_{1}}=2.02.$$

This result is very close to 2, which is the value predicted in the continuum model. Therefore, the semiclassical MM/DFT approach tends to the classical continuum macroscopic limit, proving that the main quantum contribution to the deviation from this model is due to the small change in the equilibrium geometry.

### 3.2. Quantum Revivals

We can write the initial state of a time-independent Hamiltonian $\hat{H}$ as a linear combination of its eigenfunctions:

(5) $$|\Psi (0)\rangle =\sum_{n=0}^{\infty }a_{n}|u_{n}\rangle ,$$

where $a_{n}$ are constants and $|u_{n}\rangle$ are the eigenfunctions with energies $E_{n}$,

(6) $$\hat{H}|u_{n}\rangle =E_{n}|u_{n}\rangle .$$

The temporal evolution of this state can be written as

(7) $$|\Psi (t)\rangle =\sum_{n=0}^{\infty }a_{n}|u_{n}\rangle e^{-\frac{i}{\hbar }E_{n}t}.$$

Since we have calculated the energy of curved graphene nanoflakes using DFT (i.e., solving the Kohn–Sham equations for the system) we know their energy spectrum and can therefore study the time evolution of a wave packet in these systems. Let us consider a superposition of eigenstates of the Hamiltonian concentrated around a central energy level $n_{0}$ characterised by an energy $E_{n_{0}}$. We can perform a Taylor expansion of the energy spectrum around $E_{n_{0}}$:

(8) $$E_{n}=E_{n_{0}}+E_{n_{0}}^{\prime }\left(n-n_{0}\right)+\frac{1}{2!}E_{n_{0}}^{\prime \prime }{\left(n-n_{0}\right)}^{2}+\frac{1}{3!}E_{n_{0}}^{\prime \prime \prime }{\left(n-n_{0}\right)}^{3}+\ldots .$$

Taking into account Equations (7) and (8),

(9) $$|\Psi (t)\rangle =\sum_{n=0}^{\infty }a_{n}|u_{n}\rangle e^{-\frac{i}{\hbar }\left[E_{n_{0}}+E_{n_{0}}^{\prime }\left(n-n_{0}\right)+\frac{1}{2!}E_{n_{0}}^{\prime \prime }{\left(n-n_{0}\right)}^{2}+\frac{1}{3!}E_{n_{0}}^{\prime \prime \prime }{\left(n-n_{0}\right)}^{3}+\ldots \right]t}.$$

Each term in the exponential (except the first one that is just a global phase) defines a characteristic time scale:

(10) $$T_{\mathrm{Cl}}\equiv \frac{2\pi \hbar }{|E_{n_{0}}^{\prime }|}\;\mathrm{is}\mathrm{called}classical\;time,$$

(11) TRe≡2πℏ|En0″|/2iscalledrevival time,and

(12) $$T_{\mathrm{Sup}}\equiv \frac{2\pi \hbar }{|E_{n_{0}}^{\prime \prime \prime }|/6}\;\mathrm{is}\mathrm{called}super-revival\;time$$

(see [17] for further details).

We are going to consider a Gaussian initial wave packet,

(13) $$a_{n}=\frac{1}{\sigma \sqrt{\pi }}e^{\frac{-{\left(n-n_{0}\right)}^{2}}{2\sigma^{2}}},$$

where, we have selected the central level as the fourth level above the Highest Occupied Molecular Orbital (HOMO) $n_{0}=\mathrm{HOMO}+4$ and, in order to get a sharply concentrated packet σ=0.7 so that only five levels around $n_{0}$ have a significative contribution ($a_{n}>0.001$).

The easiest way of visualising wave packet regeneration is making use of the squared modulus of the so called *autocorrelation function* that measures the overlap of the wave packet at times 0 and *t*:

(14) $${\left|A(t)\right|}^{2}={\left|\langle \Psi (0)|\Psi (t)\rangle \right|}^{2}.$$

**Figure 6:**
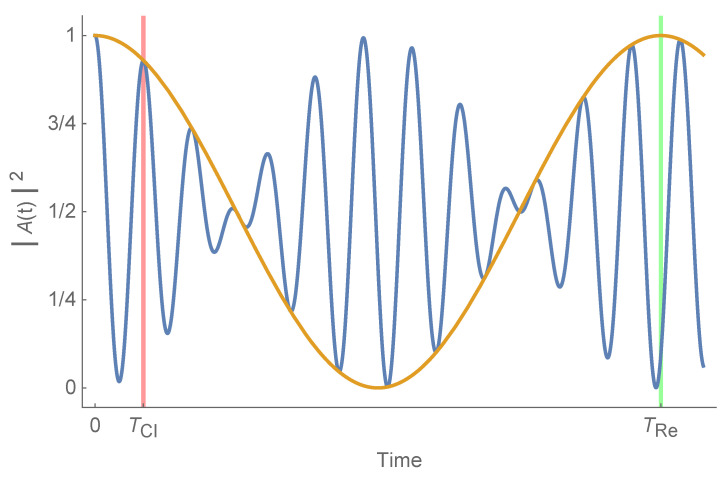
A simple example of the time evolution of the squared modulus of the autocorrelation function in blue with its upper envelope in orange. Classical time is marked in red and revival time in green.

A typical case is presented in Figure 6. ${\left|A(t)\right|}^{2}$ oscillates very fast, reaching a maximum every $T_{\mathrm{Cl}}$ inside an envelope with TRe periodicity. $T_{\mathrm{Sup}}$ is usually much larger that TRe and we will not consider it in this work.

In principle, calculating $T_{\mathrm{Cl}}$ is straightforward. One only has to search for the first maximum of ${\left|A(t)\right|}^{2}$. Determining TRe is not so easy. In this case, it is necessary to calculate the envelope of the function and calculate its first maximum. However, for some curvatures, the situation is not as clear as the one depicted in Figure 6. If TRe is not much bigger than $T_{\mathrm{Cl}}$, both times interfere, the pattern is more complicated and it is difficult to determine both of them, especially $T_{\mathrm{Cl}}$. It is then desirable to have another way of calculating these times. The solution is employing Equations (10) and (11). To do that, we have calculated an interpolating function by adjusting a parabolic curve to every three consecutive levels in the spectrum and used it to calculate the first two derivatives of the energy with respect to the level that appears in those equations. Therefore, we have two different ways of calculating these times. We will call the first one *numerical* (num.) and the second one *analytical* (an.).

Experiments for measuring quantum regeneration times are based on the use of two consecutive laser pulses [17]. The first one, called *pump*, creates the initial wave packet, while the second one, called *probe*, measures its time evolution. By changing the delay between the two pulses, different time scales can be explored. This *pump–probe* scheme was proposed by Alber, Ritsch, and Zoller [71], and was initially used to study atoms [72,73]. In this case, the second laser pulse was used to ionize the system and the photoionization signal was measured. The method was modified by Zewail (who was awarded the 1999 Nobel Prize in Chemistry for his studies in this field) and his group to study few-atom molecules, using the probe pulse to get the system to an upper fluorescent state and measure the fluorescence [74,75]. More recently, the scheme has been adapted so that the second pulse photoexcites the sample and the differential transmission spectra is analysed. This approach has made possible to study the wave packet evolution of CdSe quantum dots with a mean diameter of 6.4 nm (slightly larger than the diameter of the carbon nanoflake we have selected) [76].

#### 3.2.1. Classical Time

**Figure 7:**
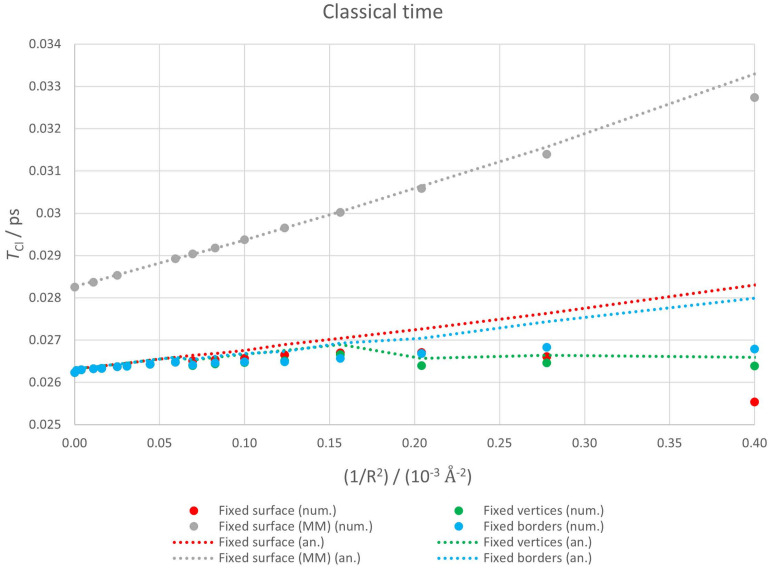
Classical times for a curved graphene nanoflake. Points correspond to numerical values while dotted lines represent analytical ones.

We present in Figure 7 classical times determined both numerically (points) and analytically (lines) for the four geometries considered: fixed surface (red), fixed borders (blue), fixed vertices (green) and fixed surface (MM) (gray). Analytical estimations are presented as lines for clarity purposes, but they can only be calculated at the same points as numerical ones. We have simply linked two consecutive points with a straight line.

The agreement between numerical and analytical estimations is good for all geometries for low curvatures (below approximately $10^{-4}Å{}^{-2}$), but in two of the three self-consistent calculations, this agreement is lost for high curvatures. The reason is that, as we will see in the following subsection, TRe decreases as the curvature increases and it becomes less that one order of magnitude bigger than $T_{\mathrm{Cl}}$ for the fixed surface and fixed border cases.

Recalling Equation (10), $T_{\mathrm{Cl}}$ is proportional to the inverse of the first derivative of the energy with respect to the level index. This means classical time is related to the spreading of the energy spectrum. $T_{\mathrm{Cl}}$ decreases as the separation among energy levels increases.

If we look at the analytical estimation for the classical times corresponding to the fixed surface and fixed border cases, they grow with curvature, while in the fixed vertices case this initial tendency breaks beyond $1.5\times 10^{-4}Å{}^{-2}$ and the graph becomes nearly flat. We have to remember that this third case corresponds to the geometry with lower restrictions and the system can adapt itself better to the deformation of its fixed points. This means that both total energy (see Figure 3) and energy level separation are less sensitive to curvature.

If we consider the numerical estimations for the three self-consistent geometries, $T_{\mathrm{Cl}}$ remains essentially flat since the slight decrease in energy spectrum spreading is compensated by the interference from TRe. In the fixed surface case this interference overcomes the tendency of $T_{\mathrm{Cl}}$ to grow and, in fact, it decreases for high curvatures.

Finally, if we concentrate on the non-self consistent fixed surface (MM) case, the behaviour is simpler. Numerical and analytical estimations agree perfectly except for the high curvature regime because, as we will see in the next subsection, TRe is much higher than $T_{\mathrm{Cl}}$. The spreading of the energy spectrum decreases monotonically with curvature and classical time increases in a linear way.

#### 3.2.2. Revival Time

**Figure 8:**
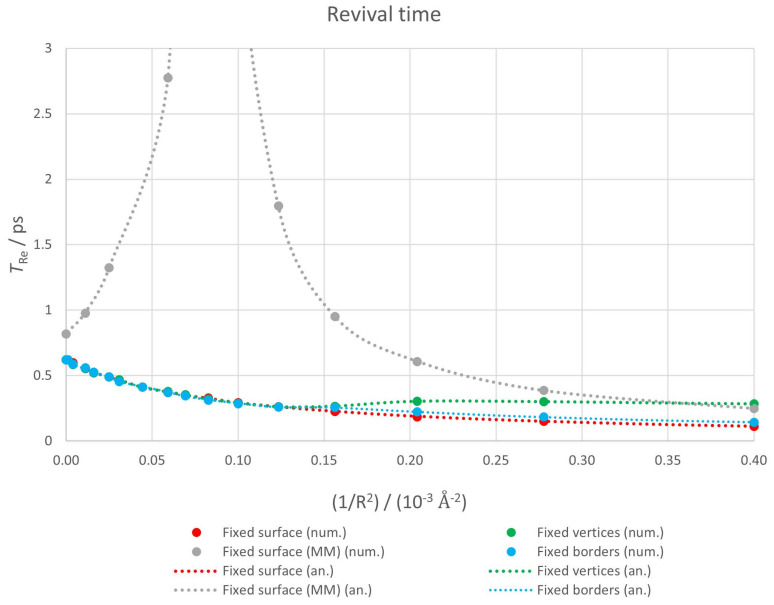
Revival times for a curved graphene nanoflake. Points correspond to numerical values while dotted lines represent analytical ones.

We show in Figure 8 revival times determined both numerically (points) and analytically (lines) for the four geometries considered: fixed surface (red), fixed borders (blue), fixed vertices (green) and fixed surface (MM) (gray). Analytical estimations are presented as lines for clarity purposes, but they can only be calculated at the same points as numerical ones. In this case, we have connected two consecutive points with a smoothed line.

In all cases, numerical and analytical estimates perfectly agree since superrevival times are much higher than revival ones and do not interfere with them.

According to Equation (11), TRe is proportional to the inverse of the second derivative of the energy with respect to the level index. This means revival time is related to the non-linearity of the energy spectrum. TRe increases as the spectrum gets closer to being linear (i.e., energy levels tend to be equally spaced).

Revival times for the fixed surface and fixed borders cases are very similar and decrease monotonically with curvature. TRe for the fixed vertices case coincides with them up to $1.5\times 10^{-4}Å{}^{-2}$ and from that point on it remains essentially constant. The situation is completely different for the fixed surface (MM) case. There is a pronounced peak around $8\times 10^{-5}Å{}^{-2}$ (in fact, TRe grows 12 orders of magnitude in this area and reaches several seconds). To check this feature is not an artifact, two of the authors wrote independently different codes (one in Scilab [77] and the other one in Mathematica [78]). The results obtained with both codes agreed.

Similar behaviours have been found in other 2D Dirac materials in external fields. For instance, planar graphene rings [26] and graphene flakes [27] in a perpendicular magnetic field or silicene in the presence of a perpendicular electric field [79]. In the latter case, the cause is a topological phase transition from a topological insulator to a band insulator at the charge neutrality point. However, to the best of our knowledge, this is the first time it appears in the absence of an external field. It is known that strain affects planar graphene revivals [24] and, as we have pointed out in the Introduction, graphene bending results in the appearance of a pseudo-magnetic field. It is possible that this pseudomagnetic field is responsible for this divergence in revival time for a particular radius. Taking into account that silicene is not a planar structure but a buckled one and that it exhibits this phenomenon when an electric field is applied, we plan to study in the future curved graphene flakes under an external electric field. This could provide a deeper understanding of the interplay between curvature effects and revival times in 2D nanostructures.

## Figures and Tables

**Table 1:** Results of the fitting of the data in Figure 5 to the Padé approximant in Equation (3).

$a_{0}$	$a_{1}/{Å}^{-1}$	$b_{1}/{Å}^{-1}$
−0.1818	0.0443	0.0219

## Data Availability

The data presented in this study is contained within the article.

## Abbreviations

The following abbreviations are used in this manuscript:

DFT	Density Functional Theory
GGA	Generalized Gradient Approximation
HF	Hartree-Fock
HOMO	Highest Occupied Molecular Orbital
LDA	Local Density Approximation
MM	Molecular Mechanics
